# Extensive Experimentations on Opportunistic Routing in Wireless Sensor Networks

**DOI:** 10.3390/s18093031

**Published:** 2018-09-10

**Authors:** Ichrak Amdouni, Cedric Adjih, Nadjib AitSaadi, Paul Muhlethaler

**Affiliations:** 1 CRISTAL Research Laboratory, National School of Computer Science (ENSI), University of Manouba, Manouba University, Manouba 2010, Tunisia; 2 National Engineering School of Sousse (ENISO), Sousse University, Sousse 4023, Tunisia; 3 INRIA Saclay, Infine Team, F91120 Palaiseau, France; cedric.adjih@inria.fr; 4 University Paris-Est, LIGM-CNRS UMR 8049/LiSSi EA 3956, ESIEE Paris, F93162 Noisy-le-Grand, France; nadjib.aitsaadi@esiee.fr; 5 INRIA Paris, Eva Team, F75012 Paris, France; paul.muhlethaler@inria.fr

**Keywords:** WSNs, opportunistic routing, low duty-cycle, LDPC, multimedia traffic, experimentation, Arduino

## Abstract

In this paper, we design and experiment ODYSSE (Opportunistic Duty cYcle based routing protocol for wirelesS Sensor nEtworks) protocol. It combines three main mechanisms: (i) duty cycle, where nodes alternate between active and sleep states, (ii) opportunistic routing where routing tables do not exist and the next hop is elected once the packet arrives, and (iii) source coding with LDPC (Low-Density Parity-Check) codes in order to face packet losses while minimizing energy consumption. We focus on two heterogeneous scenarios: bulk image transmission and infrequent events reporting. Modeling the average waiting delay of forwarders, we show that simple relay selection strategies are effective. We used 45 Arduino nodes communicating with IEEE 802.15.4 (XBee) within the large platform FIT IoT-LAB (IoT-LAB is part of the large platform FIT: Future Internet of Things). We implement and extensively study the behavior and performance of our proposal ODYSSE. We show that the three techniques fit perfectly, yielding a robust low complexity protocol for highly constrained nodes in typical IoT applications.

## 1. Introduction

The critical issue addressed in WSNs often relates to energy efficiency. Solutions have been proposed for fixed patterns of communication, such as collecting low frequency sensor measurements [1]. However, another opposite communication pattern is the bulk transmission characterising for instance the family of Wireless Multimedia Sensor Networks (WMSNs) [1,2,3,4]. The latter have shifted the focus from the typical scalar WSNs to networks which deliver multimedia traffic as video, audio, still image in addition to scalar data. The promising application fields are numerous [1], such as smart cities including areas monitoring, missing persons locating, etc.

Inspired by the new challenges of WMSNs, we propose ODYSSE (Opportunistic Duty cYcle based routing protocol for wirelesS Sensor nEtworks), a distributed and adaptative routing protocol, which can both handle infrequent and bulk traffic. Our objective is to minimize energy consumption and hence maximizing network lifetime while maintaining low end-to-end delays and overhead. Hereafter, we will introduce the main components of our proposal.

### 1.1. Duty Cycle

Applying the duty cycle for sensors [5,6] means alternating between active and sleep states while favoring the sleep state as much as possible. Obviously, such a design saves energy by avoiding idle listening, collisions and traffic overhead generation [7,8]. The duty cycle can be either synchronous or asynchronous. The first category implies clock synchronization and coordination between nodes to set their schedule. An example of framework for synchronous duty cycle is the work of the 6TiSCH (IPv6 over the Time Synchronized Channel Hopping mode of IEEE 802.15.4e) working group [9,10] from the IETF (Internet Engineering Task Force). According to 6TiSCH, communications are coordinated based on a slotted time division multiple access (TDMA) with frequency hopping. In contrast, in asynchronous design, sensor nodes wake up independently and perform “online” sender-receiver coordination.

To avoid synchronization overhead, ODYSSE is based on asynchronous sender-initiated coordination (like B-MAC [11]). To further improve delays, two adaptive variants are proposed; the duty cycle is tuned depending on the traffic (infrequent events or bulk transmissions).

### 1.2. Opportunistic Routing

The rationale behind opportunistic routing [12,13] is to exploit the broadcast nature. Indeed, when nodes which are closer to the destination overhear a transmission, they are allowed to participate in forwarding the packet. A side effect is that energy consumption may be spread more evenly over multiple paths, making also possible a forwarding strategy avoiding intermediate forwards with low battery levels. Moreover, latency is improved, along with capacity and fault tolerance.

Using an opportunistic routing strategy, one critical question is which neighboring node forwards a given packet. The answer to this question is not straightforward because: (i) nodes have asynchronous sleep schedules, (ii) data arrival time is not necessarily predefined; when multicast is used, packet duplication occurs, and (iii) finally, complex methods do not fit low capacity sensors and delay-sensitive applications. Solutions vary from selecting the first awake node [14], or conducting a more or less complex selection process [15]. For our case, our proposal ODYSSE can wait for a specific duration for potential forwarders and can select the best one with respect to many parameters such as residual energy. We will also prove through theoretical modelling that selecting the first available forwarder independently of the provided progress towards the source is the optimal choice from delay perspective under some conditions.

### 1.3. Source Coding

In wireless network communications, faulty equipment, congested routers, collisions, interferences, etc. can lead to a partial or complete loss of packets. Conventional wireless communications deploy reliability improving techniques such as full data replication or on-demand retransmission. These techniques are too expensive or even not possible due to very strict energy constraints and asymmetric wireless channels. Erasure Codes (EC) allow to enhance the reliability of data transmissions by transmitting redundant data [16,17,18,19]. Applying such methods in WSNs faces the challenge of supporting more or less complex methods for tiny sensors. A tradeoff between (i) reliability achieved, (ii) energy, and (iii) delay overhead is investigated in [16]. The result is that ECs improve the communication reliability considerably with a small delays increase. The same conclusion is proved by our experiments (see Section 5.7).

Preliminary results of ODYSSE have been partially published in [20]. In this present paper, a theoretical model and an extensive experimental evaluation are enriching [20]. The contributions of this paper are of different nature: algorithm design, theoretical modeling and extensive experimentations. More specifically, the major contributions of this work and the structure of the paper are:

1. Design of ODYSSE: a distributed and adaptive duty-cycle based opportunistic routing protocol in WSNs. To the best of our knowledge, it is the first work which combines opportunistic routing, duty cycle and source coding in real testbed (Section 3). In particular, we will show that adapting the duty cycle to the application scenario is an efficient strategy for energy conservation in WSNs.
2. Reliability enhancement based on LDPC erasure codes [21]. The erasure codes are used to enhance reliability. We will justify that increasing the code rate allows to approach 100% of reliability. As increasing the code rate is limited by the hardware capacity, we provide some guidelines to optimize the LDPC codes implementation (Section 3.7).
3. Modeling of the average waiting time for any forwarder. The finding is interesting: selecting the first awake forwarder (independently of the progress towards the source that it provides) is more delay efficient than waiting for more replies (from potential other forwarders closer to the source) (Section 4). We show that there is a match between theoretical and practical forwarders waiting time (Section 5.3).
4. Deployment of a real 45-arduino WMSN testbed in the large-scale testbed FIT IoT-LAB [22,23]. ODYSSE is implemented and evaluated using this experimental platform (Section 5). Experimental results highlight that the mechanisms that ODYSSE includes fit very well. Also, the adaptiveness of the duty cycle outperforms the basic version (infrequent events and random duty cycle).

## 2. Related Work

### 2.1. Opportunistic Duty Cycle Based Routing in WSNs

Many works [14,24,25] have applied the opportunistic routing to save energy in WSNs. Many metrics are proposed in the literature. In [24], ORW applies asynchronous low duty cycle and selects the potential forwarders according to EDC: the Expected number of Duty Cycled wakeups until a packet has reached its intended destination. However, packet duplication still be possible. In [26], authors propose ORIA protocol which is an improved version of ORW supporting in-network aggregation. The protocols proposed in [24,26] require the exchange of duty cycle information to update the EDC metric. In [14], the residual energy is used as a metric to select packet forwarders which contributes to balance energy consumption among all nodes.

In ASSORT (Asynchronous Sleep-wake Scheduling and Opportunistic Routing Technology) [25], whenever a node has data to transmit, it broadcasts beacons to collect potential forwarders. If no acknowledgement is received, the node retries to find a forwarder after a sleep period. This waiting time increases data delivery delays. In ASSORT, the routing metric is based on: residual energy, link reliability, and sleep-wake schedules. Similarly, the paper [27] considers the problem of relay selection. The objective is to reduce energy consumption and to avoid routing through nodes with low residual energy. Thus, like [14] for instance, nodes are prioritized according to two metrics: their distance to the sink and their residual energy. The authors evaluate the performance of their proposal via a real testbed using Zigbee nodes in both indoor and outdoor scenarios.

In RI-MAC (Receiver-Initiated Medium Access Control) protocol [15] when any node turns on its radio, it transmits a short beacon frame to announce that it is ready to receive data. To avoid collisions, the receiver informs its neighbors about its collision window, that is the period that must be used to communicate with it. In the same category of receiver-initiated MAC protocols, CD-MAC (Contention Detectable MAC) [28] has been proposed more recently. In CD-MAC, once a sender receives a probe from a receiver, it acknowledges it. Then, the receiver will poll one after the other each of the potential forwarders having sent such an acknowledgement. The proposed scheme has been implemented in TinyOs on TelosB motes. Unlike RI-MAC, B-MAC [11] is sender-initiated. Each node willing to send data, transmits a “wakeup signal”, called a preamble which lasts longer than the receiver sleep interval. Each node periodically wakes up to check if there is any preamble, in which case it remains active to receive possible incoming packets.

Despite its advantages, opportunistic duty cycle comes with challenges that should be faced. In particular, the tradeoff offered by these two techniques has been extensively studied. For instance, Reference [29] focuses on the throughput and the energy efficiency while considering the duty cycle in mobile wireless networks. The authors establish an analytical model to determine the evolution of these two metrics. Results show that throughout and energy consumption depend on listening durations. As many other papers, the paper ends by proving the advantages of the duty cycle on energy consumption. Reference [30] deals with another aspect in opportunistic routing which is packet duplication. Through a coordination method between a source and its potential forwarders, authors significantly reduce the amount of duplicate data. For our case, ODYSSE avoids the duplicate packet overhead and ensures packet unicity in the network. In [31] authors focus on the tradeoff between energy gain and delay loss when applying the duty cycle. They propose a method to enable nodes to dynamically adjust their duty cycle depending on the application delay requirements. The work in [32] mainly focuses on the load balancing issue in opportunistic routing. The proposal addresses this problem by considering the residual energy as a criteria to select the forwarder and by controlling the number of forwarders based on forwarding cost estimation.

When applying the opportunistic routing with the duty cycle, the multihop broadcasting becomes a very challenging problem when every node has its own working schedule. This issue is addressed for instance by Xu et al. in [33,34]. The strategy of these references is to minimize the number of broadcast transmissions while keeping a good latency. The authors start by proving that this problem is NP-hard (Non-deterministic Polynomial-time). Then, the basic idea of the proposed solution is that for each node there are two types of neighbors: those which receive the data packets immediately, and those which postpone their wakeup schedules to receive the broadcast data from the first category of neighbors. The authors model the problem as the Directed Latency-optimal Group Steiner Tree Problem (DLGST).

Another issue of opportunistic routing is addressed in [35]. Indeed, authors argue that opportunistic routing should take into account the correlation between links (the packet receptions across multiple receivers have certain correlation and are not independent). Thus, authors propose a metric by selecting the nodes with diverse low correlated links as forwarder candidates. They evaluate the proposal with testbed implementation and simulations.

### 2.2. Error Codes

An Erasure Code (EC) is a forward error correction code that, operating in the application layer [21,36], assumes that a packet is either received without errors or completely dropped. ECs enhance data transmission reliability by introducing redundancy, without the overhead of strict replication.

One major concern for EC mechanisms is the complexity of the mechanism itself. For instance, in [16], authors investigate the trade-off between reliability achieved and the energy and delay overhead for different EC techniques. They proved that ECs improve the communication reliability considerably with a small delays increase. In [17], authors conducted a theoretical study and real experiments on solar power sensors to dynamically adjust the redundancy of erasure codes regarding the energy level of sensors. The objective is to maximize end-to-end packet delivery without decreasing the network lifetime. The paper [18] focuses on body WSNs to investigate the decoding power consumption of LDPC codes [37]. The authors propose a scheme to determine the number of this code iterations to allow sensors to save energy while reaching a specific bit error rate.

In WMSNs, reliability is a major concern. A lost packet may dramatically degrade video or still image quality. In [19], authors analysed the impact of retransmission and wireless application-layer redundancy by using packet arrival probability and average energy consumption. They concluded that using the erasure code is more reliable and energy efficient than retransmission when the packet loss probability is low, but the performance of the erasure code deteriorates when high packet loss conditions occur. Similarly, authors in [38] evaluate the performance of conventional error control schemes (EC, ARQ (Automatic Repeat Request), etc.) in WMSNs as a function of the bit error rate. They find that the best strategy is to use cross-layer scheme in low bit error rates and link-layer hybrid schemes in high bit error rates.

All works in [39,40,41,42] proposed distributed erasure code methods where nodes inside the network perform coding unlike our work where only the source node needs to perform coding. The encoding scheme used differs from paper to another. For instance, in [39], authors use erasure codes to ensure data retrieval in case of node failure inside a distributed storage WSN. They use the systematic optimal locally recoverable codes proposed by [43] (locally recoverable means few symbols are encoded to obtain a new symbol). Both works in [40,41] combine Reed-Solomon-based erasure coding with selective retransmissions in order to achieve higher reliability. In particular, ref. [41] applies its scheme to under water acoustic sensor networks. Furthermore, in case of packet loss, receivers send negative acknowledgements along with information about the number of packets received correctly. This enables senders to determine which packets a receiver is waiting for, and thus to retransmit them. Retransmissions are stopped after receiving a sufficient number of packets, that is a number that enables original lost packets decoding. This condition optimizes the number of retransmissions. Al-Awami et al. [42] introduced the concept of survivability which is defined as the maximum fraction of sensor nodes that can fail without compromising the recoverability of the native data. The authors proposed an energy efficient distributed erasure codes for data survivability. Then, they enrich their initial proposal using the random linear network coding.

## 3. Proposal: ODYSSE

### 3.1. WMSN Architecture and Objectives

**Figure 1:**
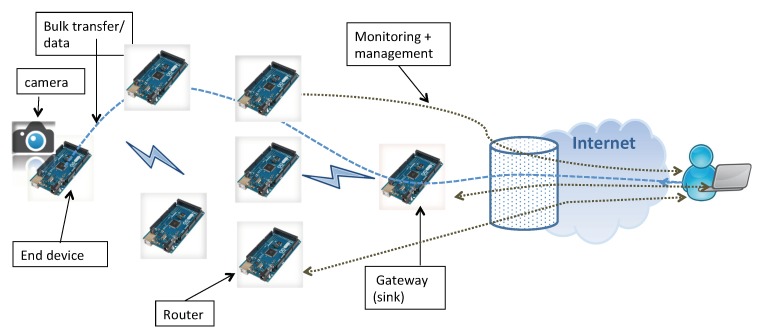
WMSNs architecture.

We consider a WMSN as depicted in Figure 1 including:

1. one multimedia end device equipped with a camera, called source, generating either low rate data or bulk data (image)
2. router devices forwarding this traffic inside the network
3. gateway device collecting data generated by the sources

One example of targeted application is recording images from the monitored area for future usage (post-analysis of data in case of accident for instance [1]). All devices are battery powered, except for the gateway which has a permanent energy power. The gateway has two interfaces: one with the network devices enabling it to collect data, and the other is linked to the end-user to allow the remote management, the monitoring, etc.

ODYSSE is responsible for routing data from the source to the gateway while optimizing the lifetime of the network. In addition, ODYSSE aims at keeping low data transmission delays and overhead since it deals with small capacity devices.

### 3.2. Overview of the ODYSSE Protocol

ODYSSErouters switch to the sleep state asynchronously and randomly. As a consequence, the wireless network topology is dynamic and unpredictable which makes the application of the classical reactive and proactive routing not practical. Thus, ODYSSE protocol adopts an opportunistic routing approach.

Unlike classical routing schemes, opportunistic routing does not build a fixed routing table beforehand. However, forwarders (i.e., next hops) are selected on the fly. To keep low complexity forwarder search method, in ODYSSE, a forwarder is a neighbor offering the best compromise for the protocol adopted policy depending on its: (i) residual energy, (ii) geographic distance relative to the gateway, and (iii) quality of wireless links. To ensure routing progress towards the gateway and to avoid routing loops at each hop, a DAG (Directed Acyclic Graph) rooted at the gateway is implicitly constructed and maintained by each node as its “distance” relative to this gateway. This distance is essentially the number of hops separating the node from the gateway but taking into account the wireless link quality by exploiting the Received Signal Strength Indicator (RSSI) metric.

The source node generates, in addition to the original data packets, repair (redundant) packets which are linear combinations of original ones. The repair packets are routed the same way as original packets. This data redundancy enables the final destination to decode the received packets in order to retrieve the lost ones. ODYSSE is a distributed protocol. As routes are computed on the fly, nodes do not need to store topological information as in classical routing protocols. ODYSSE is implemented and experimented on a large-scale experimental platform of Arduino devices under real radio conditions.

In the following sections, we will detail the main operations of ODYSSE.

### 3.3. Distance Computation

The RSSI is a relevant basis for computing link metric. In fact, it provides a good compromise between path lengths and links quality. It is also an indicator of packet loss [44].

Consequently, each link is characterized by a metric denoted “link_metric” which is defined by Algorithm 1 as follows:
**Algorithm 1**link_metric computation1:**if** (RSSI < RSSI_THRESHOLD) **then**2: link_metric = 1+γ3:**else**4: link_metric = 15:**end if**

The parameters RSSI_THRESHOLD and γ are tuned experimentally. From this algorithm, we can see that the link_metric is equal to 1 if the link RSSI is higher than a given threshold. Otherwise this link is penalized and its distance is equal to 1 + γ. The objective is to avoid “long links”, that is links which are of a bad quality (physical distance which is approximated by the RSSI is relatively high).

From this metric characterizing a given link, a distance vector protocol computes distances from the gateway as follows: The gateway initiates the process to allow each node to estimate its distance denoted (“gateway_distance”) towards this gateway. This process is based on the broadcast of a message called “Level”, sent periodically by the gateway. The latter initialises its distance to zero and broadcasts the Level message. Receiving this message from any node *v*, each node *u* performs the following steps:

1. The gateway_distance is potentially updated as illustrated in Algorithm 2:
**Algorithm 2**gateway_distance computation1:The link_metric between *u* and *v* is computed as described in Algorithm 1.2:candidate_distance:= gateway_distance of the transmitter *v* + link_metric3:**if** (*u* has not computed its distance yet) **or** (candidate_distance < gateway_distance) **then**4: *u* updates its distance accordingly: gateway_distance:= candidate_distance.5:**end if**
2. The first step is iterated for a predefined period called LEVEL_PERIOD to potentially receive Level messages from neighbors. After this period, in case the node has updated its distance, it generates and broadcasts a new Level message including its distance. Otherwise, the Level message received is not repeated.

After these steps, we find that nodes are forming a logical tree (i.e., DAG) defining their distances towards the gateway taking into account the RSSI (Received Signal Strength Indicator) values.

### 3.4. Forwarder Search and Selection

Routing is based on a greedy approach, and forwarder selection is sender-initiated. When any node *u* (either a source node or any router node) has a data packet to transmit, it broadcasts Beacon messages. A Beacon from *u* includes its gateway_distance. Any awake neighbor *v* receiving this beacon will proceed as illustrated in Algorithm 3:
**Algorithm 3** Reception of Beacon message1:**if** (*v* is closer to the gateway than *u*) **and** (RSSI < RSSI_THRESHOLD) **then**2: *v* sends a Reply message3:**else**4: the node *v* stays silent5:**end if**

The initiator *u* will repeatedly send Beacon messages (the periodicity is given by WAIT_REPLY_ PERIOD) until the forwarder search phase expires, i.e.,:

- After a predefined period of time denoted BEACON_PERIOD,
- Or, when a predefined number (MAX_NB_REPLY) of Reply messages is received.

When node *u* receives a Reply message from any node *v*, it can read the following parameters from this message:

1. Residual energy of *v*
2. Distance of *v* relative to the gateway
3. RSSI of the Reply message from node *v*

Depending on the policy (e.g., emphasis on energy, or lower latency, etc), *u* will select the most appropriate forwarder. This design allows ODYSSE to support different policies. In our experiments, we used a policy purely based on: (i) the distance of *v*, (ii) the RSSI and, (iii) the residual energy. Each potential forwarder is characterized by a weighted sum of these three metrics (the residual energy is expressed in terms of total number of sent and received messages). The best forwarder is the one having the best sum. Once the forwarder is selected, the node *u* transmits its packet. If no forwarder is found after BEACON_PERIOD, this period is extended, unlike ASSORT [25] for instance where nodes would rather return to the sleep state. However, our approach tends to minimize delays, and we believe that it is more efficient for dense networks.

The advantages of this design are the low storage required and a light-weight selection procedure. Furthermore, ODYSSE, unlike ORW (Opportunistic Routing in Wireless sensor networks) [24] is loop free. Notice that ODYSSE does not result in redundant transmissions which is a real issue in opportunistic routing. By selecting a unique forwarder on the fly at each hop, ODYSSE guarantees packet unicity in the network (unlike ORW [24] for instance). If a node is not an intended destination of a given packet it simply ignores it. The path diversity and hence the load balancing which characterize the opportunistic routing are not compromised. Nodes alternate between active and sleep state, thus relay nodes at each hop are not always the same (with the detail that nodes close to the source are more solicited) (see Figure 12 for the average percentage of sent data packets). Also, all nodes still profit from good duty cycle allowing them to save energy (will be highlighted in Figure 11). Section 5.5 will explain these properties.

### 3.5. Duty Cycle

#### 3.5.1. Principles

In ODYSSE, source nodes and gateway are always active while routers are duty cycled. The maximum duration of the active state is fixed, while the sleep period follows a random uniform distribution. The detailed functioning of a node, starting from the active state, is described hereafter:

- IF the node is idle (has no data packet to send), it waits for Beacon messages:
  - IF after ACTIVE_PERIOD (the maximum active period of routers) no Beacon message is received, it returns to the sleep state.
  - Otherwise, IF the node has replied to a Beacon, it waits for a Data message, and then:
    ∗ IF it does not receive a Data message after WAIT_DATA_PERIOD, it returns to the sleep state
    ∗ Otherwise, it will forward the Data message as follows:
      · IF the node has data to send: it sends Beacon messages periodically. It collects replies as described in Section 3.4, until a forwarder is selected and then the Data packet is successfully sent (receiving MAC layer acknowledgements). After that, the node has no data packet to send and returns to the sleep state.

#### 3.5.2. Sleep Duration

In ODYSSE, nodes are not synchronized. They select a random sleep period within the interval I given by:

(1) I=[MIN_SLEEP_PERIOD,α×ACTIVE_PERIOD]

where MIN_SLEEP_PERIOD and ACTIVE_PERIOD are some of ODYSSE parameters. We will vary α in experimentations. The random sleep period is actually adjusted in some cases, according to two application scenarios:

1. Infrequent events: This classical WSNs scenario is characterized by low volumes of data generated either periodic, regular, rare or exceptional. With this assumption, router nodes have just to choose a sleep period within the I interval. This is because in this scenario, a current packet is rarely a predictor for an additional incoming packet. This algorithm will be denoted INFR.
2. Multimedia flows: This scenario considers a bulk data transfer of still images from the source. For this scenario, we define two algorithms:
  (a) MED_ADAP (MultimEDia ADAPtive): routers adapt their duty cycle by assuming existing traffic and shortening their duty cycle. Indeed, when any router transmits a packet, instead of turning back to the sleep state for a random period, it rather sleeps for the minimum period MIN_SLEEP_PERIOD for a predefined number of times (SHORT_SLEEP_COUNT). That is, before SHORT_SLEEP_COUNT packets transmitted, the node sleeps a minimum period of time between two transmissions. After that, it returns to the normal behavior (the sleep period is a random period in the interval I). With this adaptive strategy, the network will face the congestion resulting from the bulk transfer. We will show in Section 5.4 that this method significantly reduces end-to-end delays without compromising the energy saving.
  (b) MED_N_ADAP (MultimEDia Non ADAPtive): This scenario is the same as the MED_ADAP, but nodes do not adapt their duty cycle, they select a random sleep period within the I interval.

### 3.6. Example of ODYSSE Operations

**Figure 2:**
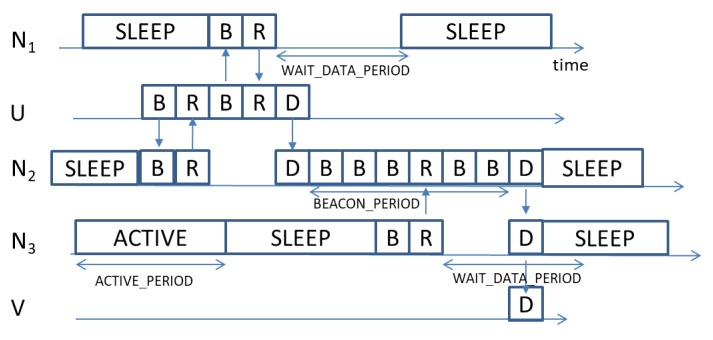
Illustrative example for data routing in ODYSSE. The node U is a source node sending a packet to node V. The node U has as neighbors nodes $N_{1}$ and $N_{2}$. Node $N_{2}$ has as neighbors nodes U and $N_{3}$, while $N_{3}$ has as neighbors nodes $N_{2}$ and V. Node V has as neighbors node $N_{3}$. Messages are denoted: “B”: Beacon, “R”: Reply, “D”: “Data”, “SLEEP”: sleep period, “ACTIVE”: active period.

**Figure A1:**
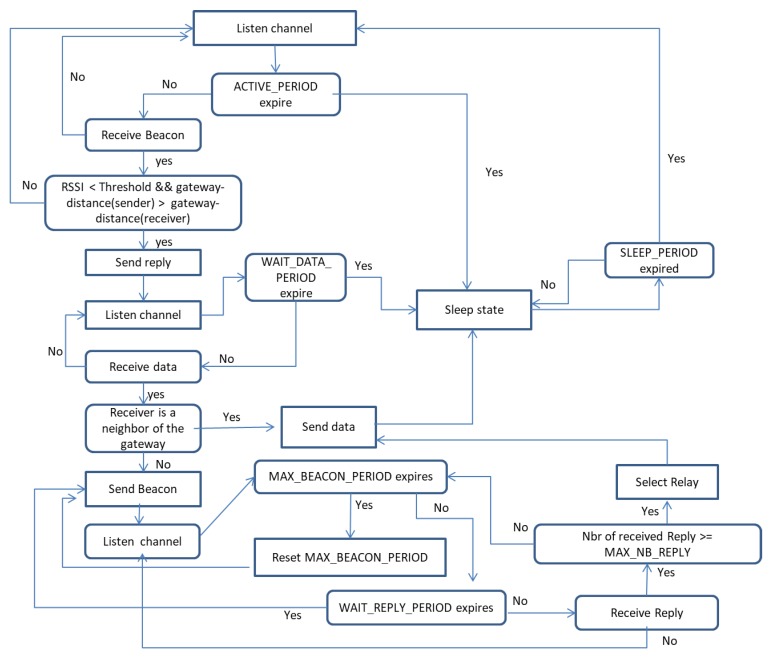
The flowchart of ODYSSE.

Figure 2 illustrates the general functioning of ODYSSE. The source U has a data packet “D” to send to node V. It starts by transmitting beacons “B”. We set the parameter MAX_NB_REPLY to 2, hence node U should receive two Reply messages before selecting the forwarder. Node $N_{2}$ replies “R” before node $N_{1}$ because this latter was in the sleep state. Node $N_{2}$ remains in the active state for a maximum period given by WAIT_DATA_PERIOD. Meanwhile, the node $N_{1}$ becomes active and receives the beacon message from U, thus it sends back a Reply message, then it waits for WAIT_DATA_PERIOD. Receiving two Reply messages, the source U selects node $N_{2}$ as a forwarder and transmits the data message. At node $N_{1}$, the WAIT_DATA_PERIOD expires with no data received, it then returns to the sleep state. Node $N_{2}$ starts sending beacons. The node $N_{3}$ replies when it wakes-up. However, as the MAX_NB_REPLY is set to 2, node $N_{2}$ waits the end of the MAX_BEACON_PERIOD to transmit the packet “D” to node $N_{3}$. Finally, node $N_{3}$ forwards the data to the final destination V. A flowchart illustrating the operations of ODYSSE is provided in the Appendix (Figure A1).

### 3.7. Packet Erasure Codes in ODYSSE

This section describes how ODYSSE is able to combat wireless transmissions losses.

Because ODYSSE operates at the routing layer, our focus is on the packet level rather than the bit level. Thus, we consider, as in RFC 5170 [36], a packet erasure channel, that is a communication path where packets are either dropped or received. When a packet is received, it is assumed that it is not corrupted. Consequently, ODYSSE considers Erasure codes (EC) [38], which are based on data redundancy. Indeed, instead of sending *k* original packets, the encoder adds *m* redundant packets to recover the lost packets. When the number of the received packets is sufficiently high, the decoder decodes them and retrieves the original packets. Different coding methods exist. In our work, we rely on the LDPC (Low-density parity-check) codes [37]. The latter is a block code defined by a sparse parity-check matrix (majority of entries are zero), and is known to provide excellent decoding performances [45].

#### 3.7.1. Preliminaries

The terminology is the same as the one from RFC 5170 [36]. Packets are supposed of equal size and are considered as vectors of GF(2), i.e., a Galois field of two elements (a Galois field is a field that contains a finite number of elements). We depart from some traditional presentations, as in our context the symbols are actually these packets. Hence obviously vectors of symbols can be represented as matrices. Consider a source node having *k* original packets, which can be considered as row vectors of a matrix, denoted *S*. Along with the *k* source packets, *m* additional repair packets are generated yielding n=k+m packets (systematic codes). The matrix representing all packets (source and repair packets) is denoted *P*.

In general, LDPC codes (systematic or not systematic) are linear mappings: *P* is obtained as $P=G^{T}S$, where *G* is a generator matrix. By definition of LDPC, the code has one parity check matrix *H*, and the generator matrix must satisfy $HG^{T}=0$ and hence HP=0.

For decoding, the method is to recover the source packets from the received coded packets: because of the linearity, we have just to solve the linear equations induced by HP=0, where the unknown are the source packets while the constants are the received ones. This could be solved by Gaussian Elimination, but for LDPC codes, more efficient, algorithms exist [45].

#### 3.7.2. Practical Implementation of LDPC Codes

**Figure 3:**
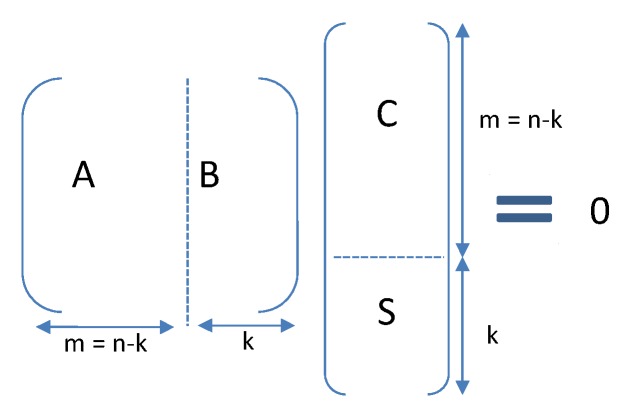
Relation between parity check matrix and generated packets.

In ODYSSE, the error correction works as follows:

1. We use systematic coding in which the original source packets *S* are sent uncoded: *S* is a submatrix of *P*. For simplicity of exposition, we assume in the following that the source packets follow the repair packets (in the matrix), hence the matrix *P* is composed of m=n−k repair packets denoted *C* followed by *k* source packets *S* (see Figure 3).
2. Thus as $P=G^{T}S$, the last *k* columns of *G* will then be the identity matrix. Also, because $HG^{T}=0$, it follows that *B* is equal to the identity. We have then: $C=-A^{-1}S$.
  Consequently, to generate the repair packets, ODYSSE pre-computes $A^{-1}$, the inverse of *A*, submatrix of *H*, the parity-check matrix. Notice that LDPC code is sparse. However, the inverse is not necessarily sparse. It is worth taking this into consideration because the less the matrix is sparse, the more memory required to store its coefficients.
  Binary LDPC codes are used, that means that packets are vectors of GF(2); addition corresponds to exclusive-or (XOR) operation on original packets *S* (and −x=x). In our case, we use the LDPC creation algorithms and source code from [46] to generate the matrices.
3. To generate the repair packets, one trivial solution would be to store all the original *k* packets and then apply $C=-A^{-1}S$. This requires a memory and storage overhead that usually does not fit low capacity sensors. Thus, ODYSSE stores instead the repair packets and updates them incrementally. Each time such a source packet $S_{j}$ is generated (j=1…k), all the repair packets $C_{i},\left(i=1\ldots m\right)$ where $S_{j}$ appears are updated: $C_{i}=C_{i}\;+\;S_{j}$ (‘+’ corresponds to a XOR).
4. Repair and original packets are routed identically.
5. At the receiver side, the decoding is done starting from the relation $C=-A^{-1}S$. This relation implies several linear equations that may be solved. Zero, some or all of the lost source packets will be recovered, depending on the losses.

Because the code is systematic, at least all source packets that are not lost, are available.

## 4. Estimation of Forwarder Search Delays

The critical question in opportunistic routing is how to choose the forwarder and how long a node should wait for potential forwarders. We can identify a tradeoff: waiting short time would decrease delays, but for applying QoS routing metrics (such as considering energy efficiency), a sufficient number of replies should be collected. In this section, we answer the question “how long shall nodes wait for potential forwarders” while focusing uniquely on the delay point of view. We elaborate a simplified theoretical model and we proceed as follows:

- We determine the average progress towards the final destination when taking the first available forwarder.
- We determine the delay between the *k*-th and the (*k* + 1)-th available replies (assuming exponential and uniform distributions of wake-up intervals of nodes.)
- We will show that selecting the first available forwarder is a delay saving strategy.

In the rest of this section, we detail these steps.

### 4.1. System Model and Notations

Consider a node that route data to the first available forwarder. This forwarder is the first node that is closer to the destination. As in [47,48] (see their Section 3.3.1), the wake up time and the position of the forwarders are independent. However, a difference is that we will consider an asymptotic model where the destination is assumed to be at infinity, the network density is uniform, and the network infinite. We assume that the radio range is normalized and is considered to be equal to 1. We consider one step: a node at position (0,0) and the destination at x=+∞,y=0.

### 4.2. Average Progress When Selecting the First Forwarder

The forwarder node will be the first one waking-up inside the half-disk of center (0,0) with radius 1 with x≥0. Its position is a random variable $X_{1}$. Assuming that nodes are distributed uniformly and since wake-up time is independent from positions, the density of probability of the random variable $X_{1}$ is: $f_{1}\left(x\right)=\frac{4}{\pi }\sqrt{1-x^{2}}$. The average value of $X_{1}$ is:

$$\begin{array}{ccc} E(X_{1}) & = & \int_{t=0}^{t=1}tf_{1}\left(t\right)dt\\ & = & {\left[-\frac{4}{3\pi }{\left(-x^{2}+1\right)}^{\frac{3}{2}}\right]}_{0}^{1}\\ & = & \frac{4}{3\pi } \end{array}$$

This is the average amount of progress per hop towards the gateway when selecting the first forwarder (expressed in “radio range” units).

### 4.3. Discussion on Waiting for the k-First Replying Forwarders

As discussed in [48] for instance, the times, at which the *k*-first forwarders will reply, corresponds to the order statistics [49] of the random wake-up time of nodes. If a node has *M* possible forwarders, the time at which they will wake-up is given by the *M* random variables $Y_{1},\ldots ,Y_{M}$. The sorted sequence of these wake-up times is the *order statistics* and is denoted $Y_{\left(1\right)}\leq Y_{\left(2\right)}\leq \ldots \leq Y_{\left(M\right)}$. Now, we compute the average value of $Y_{\left(k\right)}$ in two specific, but largely applicable, cases:

- IF each of the $Y_{i}$ is exponentially distributed, THEN:
  $$\begin{array}{c} E\left(Y_{\left(k\right)}\right)=\sum_{i=M-k+1}^{i=M}\frac{1}{i}=\frac{1}{M}+\frac{1}{M-1}+\ldots +\frac{1}{M-k+1} \end{array}$$
  Furthermore, because even for dependent variables *U* and *V*, we have E(U−V)=E(U)−E(V), therefore the mean delay between the *k*-th and (k+1)-th reply is:
  $$\begin{array}{c} E\left(Y_{\left(k+1\right)}-Y_{\left(k\right)}\right)=E\left(Y_{\left(k+1\right)}\right)-E\left(Y_{\left(k\right)}\right)=\frac{1}{M-k} \end{array}$$
  It results that for instance, $E\left(Y_{\left(2\right)}\right)-E\left(Y_{\left(1\right)}\right)=\frac{1}{M-1}$ and we have $E\left(Y_{\left(1\right)}\right)=\frac{1}{M}$. One fundamental result is that $E\left(Y_{\left(2\right)}-Y_{\left(1\right)}\right)>E\left(Y_{\left(1\right)}\right)$, hence a node will wait on average (slightly) longer for the 2-nd reply than from the first. Intuitively, the difference comes from the fact that, after the first reply, there are only M−1 nodes which could reply. Naturally, if *M* is large, the mean delay between the first and the second reply is small.
  **Remark** **1.** The distribution of $Y_{\left(k+1\right)}-Y_{\left(k\right)}$ is even given in [50], it is exponentially distributed as: $\mathrm{Exp}(\frac{1}{M-k})$.
- IF each of the $Y_{i}$ is uniformly distributed in [0,1], THEN the order statistic $Y_{\left(k\right)}$ has a beta distribution (using here the notation n=M):
  $$\begin{array}{c} Y_{\left(k\right)}∼\mathrm{Beta}\left(k,n+1-k\right) \end{array}$$
  and then: $E\left(Y_{\left(k\right)}\right)=\frac{k}{n+1}$. Thus: $E\left(Y_{\left(1\right)}\right)=\frac{1}{n+1}$ and $E\left(Y_{\left(k+1\right)}-Y_{\left(k\right)}\right)=\frac{1}{n+1}$. This means that a node will wait on average as long for the second reply after having received the first, as it will wait for the first reply.

### 4.4. Conclusions on k-First Forwarder Selection

These results mean that the general strategy of forwarding to the first node which replies is a viable default one:

- if transmission and then beacon initiation (forwarder search procedure) is considered to have zero delay (negligible).
- if the next-hop has itself as many (or more) potential next-hops as the current node.

Indeed the rationale behind that is: instead of having a node *A* waiting for a second reply from node *C*, it can forward to the first node *B* that replies. Then, the node *B* that replied can send a beacon to find a next-hop of itself. On average the node *B* will get a first reply from a node *D* at least as fast as the node *A* will get the reply from node *C*. Furthermore, on average (as the scheduling is independent from positions), *D* will be closer to the destination than *C*.

## 5. Testbed Deployment and Experimentations

### 5.1. Testbed Hardware

Our experimental testbed is composed of 45 Arduino nodes deployed in an area of 65×10 m. Each node has two components: (i) ATmega2560 [51]: a 8-bit microcontroller (256 KB of flash memory and 8 KB of SRAM), and (ii) XBee radio module [52] (IEEE 802.15.4). The transmission power is set to 2 dBm. Source nodes are in addition equipped with TTL serial JPEG camera [53]. The testbed is integrated in the existent large-scale platform FIT IoT-LAB [22,23], as described in [54,55]. The choice of the Arduino platform was motivated by many reasons. From one hand, this type of platform is widely used among makers and scientific community. Furthermore, Arduino platform offers the possibility to connect hardware extensions, such as still image camera (that we are using), wireless sensors, UART (Universal Asynchronous Receiver Transmitter) connection to other IoT-nodes (such as WiFi-enabled IoT ESP8266 nodes providing secure MQTT (Message Queuing Telemetry Transport) connections to the cloud), etc... From the other hand, the integration of the platform within FIT IoT-LAB makes it available for other users. Even if there are other type of nodes which may be more powerful than Arduino nodes, using Arduino in our work let us face the challenge of optimizing our development and making an interesting return of experience.

### 5.2. Experiment Settings

**Figure 4:**
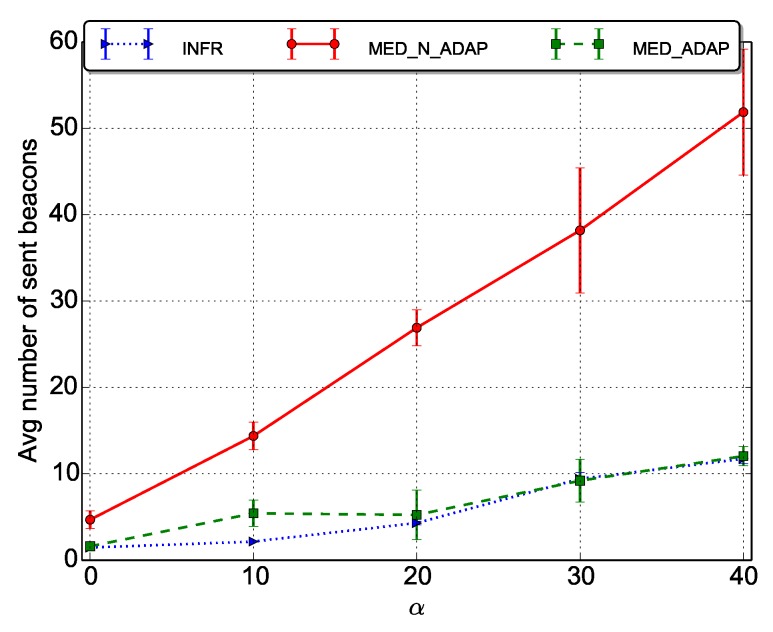
Average number of beacons sent per data packet per node.

**Figure 5:**
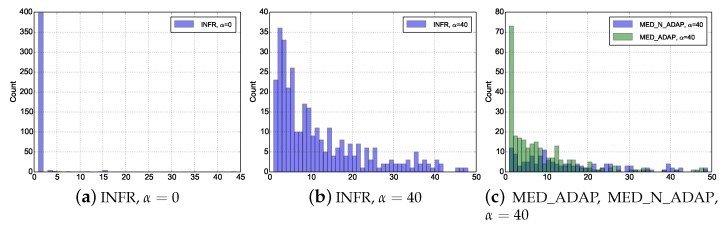
Distribution of the number of sent beacons.

**Figure 6:**
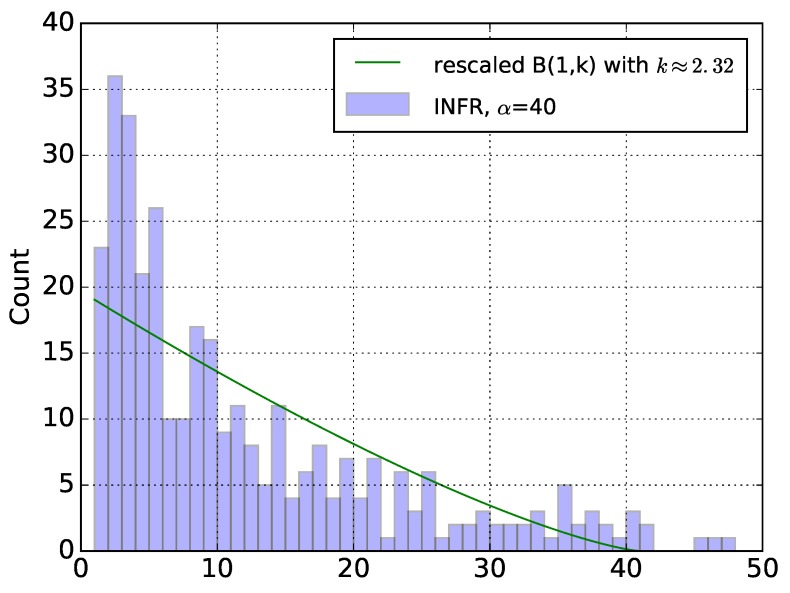
Distribution of the number of sent beacon with the rescaled Beta distribution.

**Figure 7:**
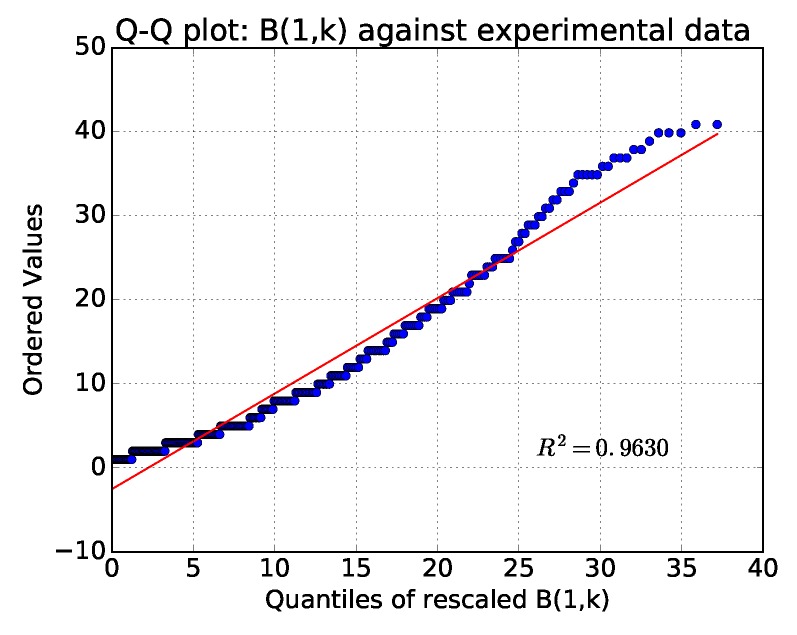
Comparing experimental distribution of delays with theoretic model distribution, through Q-Q plot.

**Figure 8:**
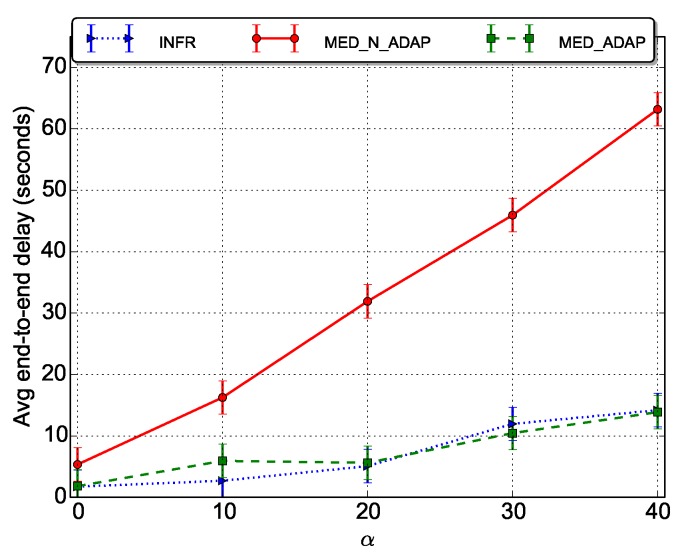
Average end to end delays per data packet.

**Figure 9:**
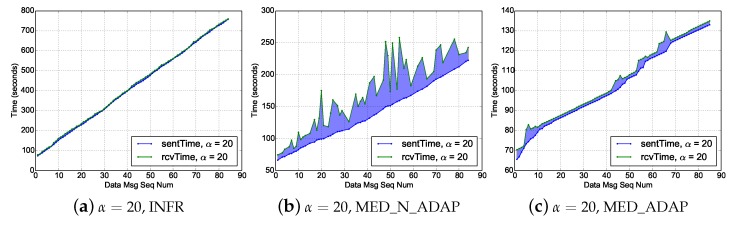
Time of sending and receiving of packets of a sample photo.

**Figure 10:**
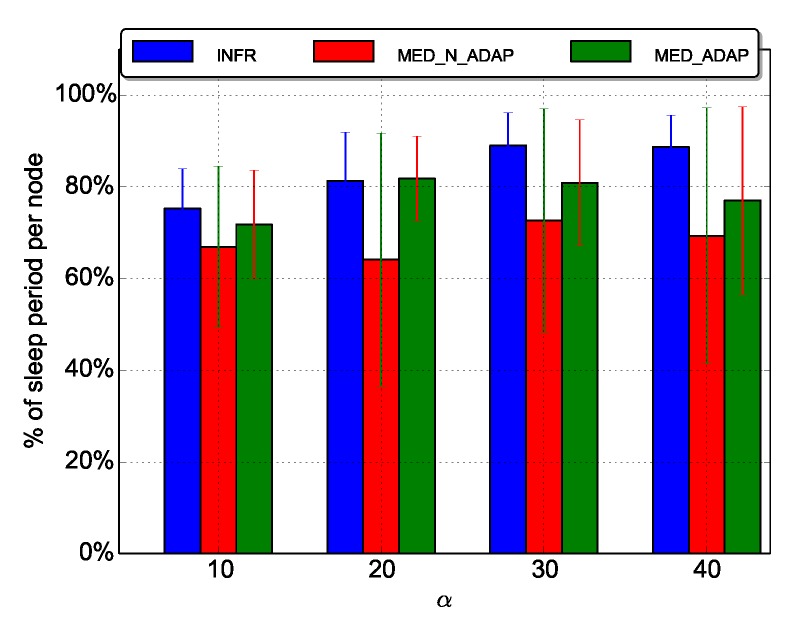
Average duty cycle per node.

**Figure 11:**
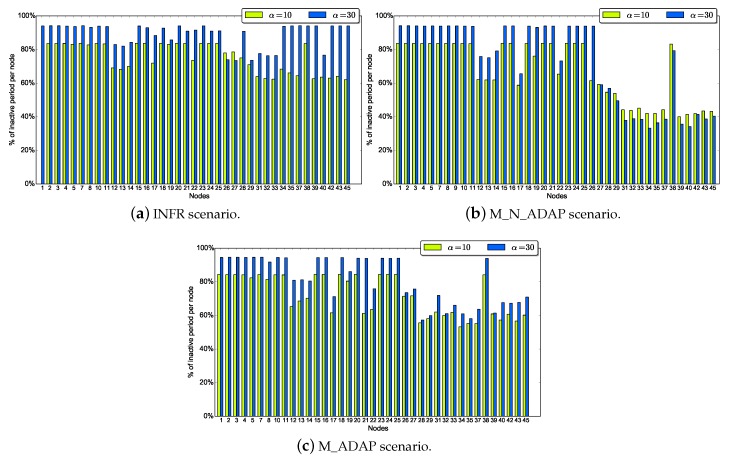
Individual duty cycle.

**Figure 12:**
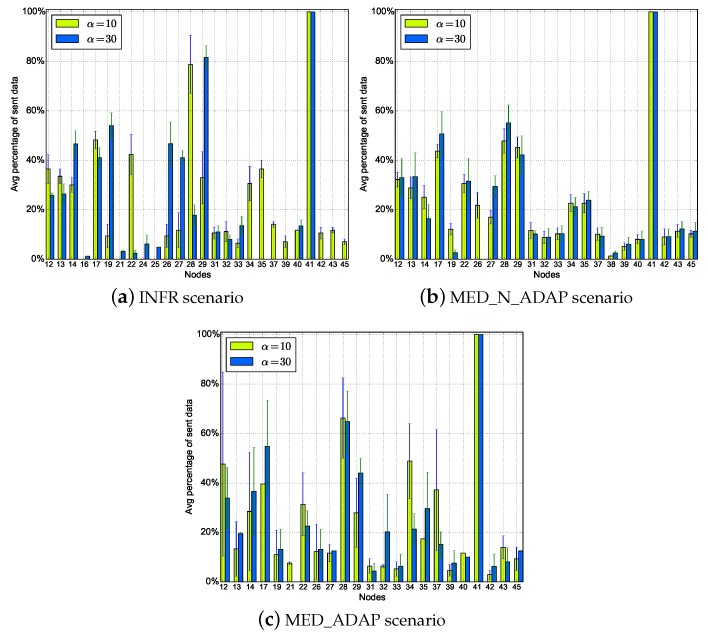
Average percentage of sent data packet per node.

The source node generates photos periodically (every 30 s). In our experiments, we vary the value of α (α=0: no duty cycle is applied) and the data redundancy β=m, that is the number of repair packets sent by the source. We mainly focus on the impact of the duty cycle and the source coding in the performance of the protocol. Thus, we vary the value of α while β is a constant and vice versa. For Figure 4, Figure 5, Figure 6, Figure 7, Figure 8, Figure 9, Figure 10 and Figure 11, the source coding is not a variable. While in Figure 12, α is set to 10. For the INFR scenario, the source generates data packets representative of infrequent traffic, with a random inter-packet delay ranging from 5 to 10 s. The other experiment parameters are summarized in Table 1. Results presented in this section are the average of 2 to 8 images.

Our result analysis originates from detailed logs collected through the serial output of Arduino devices. Python tools retrieve, store, parse, and analyse the logs (collected logs represent a volume of 26 GBytes). The physical and radio topology are described in [55].

A video illustrating a demo for ODYSSE is available online [56].

### 5.3. Overhead of ODYSSE

We evaluate the number of beacons sent per data packet per node when varying α.

As expected, results depicted in Figure 4 show that the overhead increases with α (less forwarders are available), and it is almost linear. However, the increase is more noticeable for MED_N_ADAP scenario, because in this scenario, no particular measure is applied to compensate the duty cycle of nodes. This result, actually, confirms the traditional trade-off concerning energy consumption between: (i) the duty cycle that tends to save energy, and (ii) the forwarder discovery that becomes longer and hence more beacons and energy is consumed for high values of α.

Now, looking more in details, we can analyse the distribution of the number of beacons for a sample image (Figure 5).

The immediate observation is that, with INFR and α=0, each node can immediately find a forwarder as in Figure 5a, almost after sending one beacon. More interesting, is the result with low duty cycle (high α) in Figure 5b: it appears that the distribution is more varied. The number of beacons is expected to be proportional to the waiting time given by the Beta distribution Beta(1,n) presented in Section 4 (where *n* is the number of admissible forwarders of a node).

We verify the qualitative match between the theory and our practical experiment results as follows:

- We first translate and rescale our experimental data, so that the random wake-up time of the nodes in the experiment (accounting for α, ACTIVE_PERIOD, etc.) matches best the random uniform distribution [0,1] of the model.
- Then we search for the parameter *n* which makes the distribution B(1,n) the best fit against the experimental data. For the example of Figure 5b, we find that the best fit is for n≈2.32…,
- Finally, we plot the theoretically expected histogram (by rescaling back the beta distribution B(1,n)) in Figure 6.

We can see that there is a qualitative match. To provide a more sound comparison between distributions from experimentation and model, we represented their q-q plot one against another in Figure 7: the match is satisfying.

Notice that differences are expected for a variety of reasons, such as different number of potential forwarders of different nodes, packet collisions and losses, imperfect timings in actual hardware, etc. We also note that the network behaves as if almost 2.32… forwarders are available for each node, which is rather on the low side. However, please note that losses/collisions and high number of neighbors of the sink can only decrease this number.

Figure 5c illustrates the adaptiveness of MED_ADAP scenario since the most probable number of beacons necessary to find a forwarder is 1, in stark contrast with MED_N_ADAP scenario.

The low efficiency of MED_N_ADAP compared to INFR can be noted (e.g., through the lower probability to find a forwarder with a low number of beacons, closer to 1). The explanation is as follows: in both scenarios, the nodes able to find forwarders faster are nodes with a larger number of neighbors. However, in MED_N_ADAP the network saturation is reached. In consequence, even if forwarders are found faster, forwarders themselves have to actually forward the packet to the next hop, before being available again: the benefits of having a large number of neighbors (favoring low delay) are thus naturally cancelled by congestion.

### 5.4. Average End-To-End Delays

As depicted in Figure 8, the delay plots follow the beacon overhead plots (see Figure 4) as the beaconing process is basically a source of packet forwarding delays. The end-to-end delays vary linearly with α. The scenarios MED_ADAP and INFR have low end-to-end delays that slightly increase with α. This witnesses a good trade-off between energy saving and delay reduction. In MED_N_ADAP, delays are rapidly increasing with α.

A good visualisation to understand the packet delay sources is illustrated in Figure 9 presenting the times for sending and receiving packets of a sample image for α=20. Obviously, the colored area represents the source of end-to-end delays. In MED_N_ADAP scenario, plots have high amplitude, spikes correspond to some routers that are blocked because of either network congestion or duty cycle. As explained above, these facts are alleviated by the duty cycle adaptation (in MED_ADAP) and the traffic regularisation (in INFR).

In general, while the traffic regularisation is dependant on the application (not possible in multimedia scenarios in particular), the duty cycle adaptation is an effective method to reduce delays. Also, as we will see hereafter, this adaptability is of a less impact on the energy consumption.

### 5.5. Duty Cycle

Figure 10 depicts the average duty cycle (sleep time) per node.

Selecting a random sleep period in the I interval, with the parameters of Table 1 and for α=10 for instance, any node would sleep an average period approximated by random(50,200×10)≃1025 ms, in MED_N_ADAP. Which leads to a sleep ratio equal to: $\frac{1025\times 100}{1225}=83\%$. Experiments yield an average value ≃70%.

Another observation from Figure 10, is that, as expected, INFR scenario ensures the highest values of the duty cycle. The average duty cycle increases with α for INFR scenario, while it is almost insensitive to α for MED_N_ADAP and MED_ADAP. We evaluate the duty cycle of each node in Figure 11 for α=10 and α=30.

α=30 is globally ensuring higher duty cycle than α=10. However, increasing α means that less neighbors per node will be awake which increases forwarder search phase and leads to lower duty cycle. This explains the fact that α=10 is more energy effective than α=30 for some nodes in MED_N_ADAP scenario. In contrast, in INFR and MED_ADAP scenarios, the duty cycle increases with α almost for all nodes. This means that regulating traffic injection (INFR scenario) and tuning the duty cycle (MED_ADAP scenario) allow the network to take advantage from high sleep durations setting. To conclude, high sleep periods is essential for energy saving, but adapting it to the network state is generally more effective.

Another observation from these figures is that nodes have heterogeneous duty cycles; nodes close to the source have low duty cycle compared to other nodes as their chances to receive a packet from the source when they turn on their radio are relatively high. Also, from Figure 12, we see that even nodes with high activity reach good duty cycle.

### 5.6. Evolution of the Number of Packets in the Network

**Figure 13:**
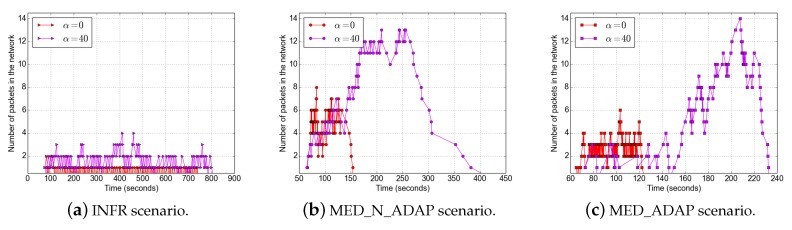
Evolution of the number of packets in the network.

Figure 13 depicts the evolution of the number of packets in the network, that is the total number of packets that nodes are buffering for transmissions.

We compare two values of α: 0 and 40. In the INFR scenario, the number of packets in the network oscillates very much; as the packets generation rate is low, their total number rapidly decreases in the network (maximum number is equal to 4 packets distributed over all nodes). The plot with α=40 reaches higher amplitudes and higher width than the plot with α=0 in all scenarios. The difference in amplitude is due to the network congestion and to the duty cycle. However, the number of packets is the network oscillates less in MED_N_ADAP scenario and it reaches its maximum values before starting to decrease. This means, as highlighted above, that some packets are more blocked in this scenario than in MED_ADAP scenario. Also, higher values of α increase the packets sojourn time in the network leading to higher end-to-end delays; which is shown by the larger width of the plots for α=40.

### 5.7. Error Correction

**Figure 14:**
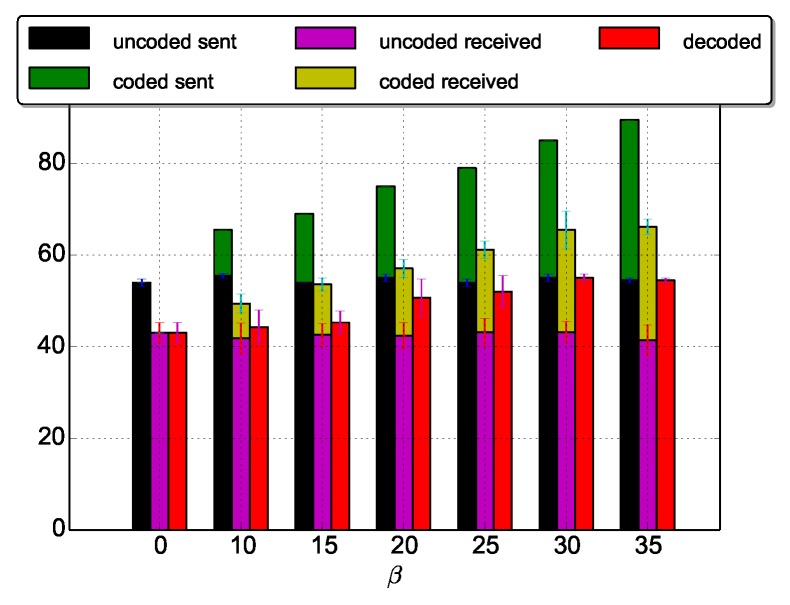
Decoding performance. The decoded packets is the total number of packets retrieved at the gateway that will be reassembled to re-constitute the image.

In erasure codes, the coding rate defined by the ratio between the number of uncoded packets and (the number of uncoded packets + the number of coded packets) is a crucial parameter as it determines the redundancy introduced in the network. Thus, we evaluate in Figure 14 the impact of the parameter β (number of coded packets). We base our experiments on the MED_N_ADAP scenario as it is a trivial solution for a challenging WSN scenario. α is set to 10. We vary the redundancy levels (0 means no redundant packets are sent). Results are the average of 8 images of the same size.

**Figure 15:**
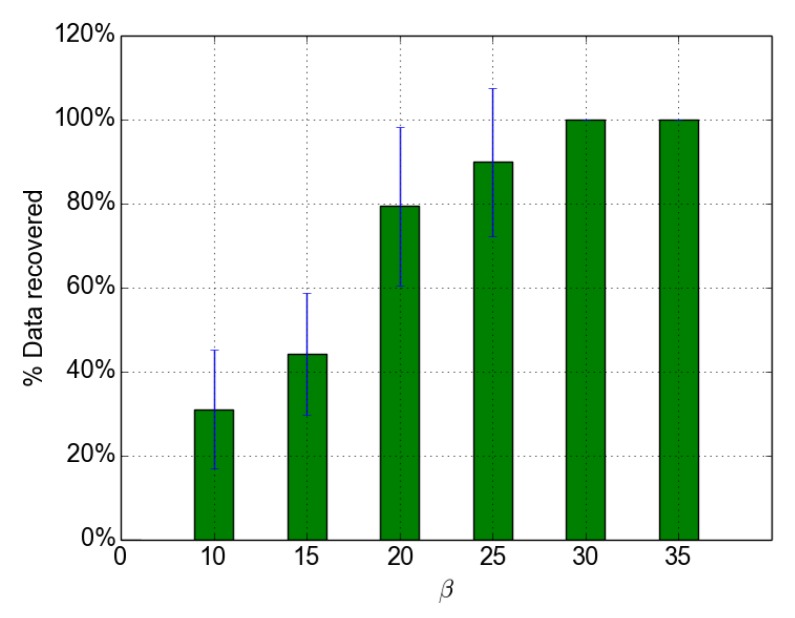
Percentage of data packets recovered.

From Figure 14, we can notice two main results. First, the number of decoded packets is higher than the number of received packets. This highlights the added value of the LDPC codes used in ODYSSE. With image transmissions for instance, losing some jpeg packets may prevent its decoding; the image cannot be displayed. Second, we notice that, the higher β is, the higher the number of decoded packets is: when β≥ 30, 100% of lost packets are recovered (see Figure 15 which depicts the percentage of recovered packets).

Please note that despite our optimized implementation of LDPC code as discussed in Section 3.7, we were limited in the maximum value of β. Our experience witnesses the importance of the optimized erasure codes.

**Figure 16:**
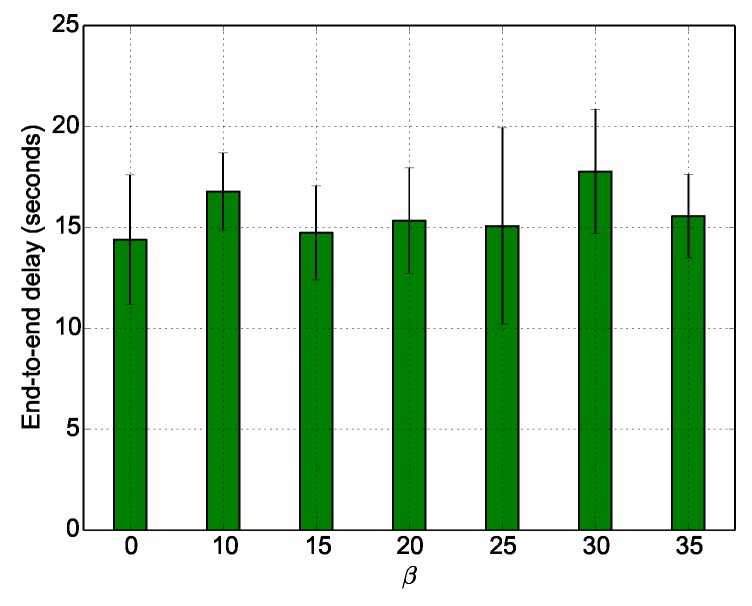
Average end to end delays per packet per data redundancy.

We now evaluate the cost of the erasure codes in terms of delays and duty cycle. Figure 16 shows that the end-to-end delays slightly varies with β. This means that coded packets do not create a particular congestion phenomena in the network compared to uncoded packets. Thus, they do not contribute to high variations of the average end-to-end delays per packet.

**Figure 17:**
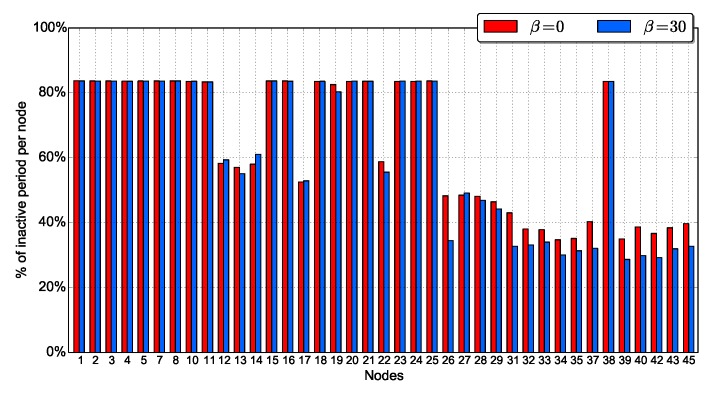
Average duty cycle per node as a function of β.

Figure 17 plots the duty cycle of nodes for β=0 and β=30. The usage of the erasure code inevitably decreases the duty cycle as nodes have to transmit more packets. However, this increase is small and mainly visible at nodes close to the source; the overall energy consumption of the network is almost constant. Notice that in ODYSSE, the coded packets computation is done only at the source, which reduces the computational energy consumption. For comparison, our findings are coherent with those in [16] which claims that the erasure codes have notable reliability improvement at a small energy and delay cost.

## 6. Conclusions

This paper provides a good reference for WMSNs studies as it reveals the behavior of sensors under real wireless conditions and real application scenarios. We proposed ODYSSE, a routing protocol tailored to fit infrequent event reporting and bulk transmissions in WSNs. ODYSSE combines three main elements: duty cycle, opportunistic routing and source coding as a reliability enhancement method. We conducted extensive experimentations with a real testbed based on Arduino platform. Results show that adapting the duty cycle to network traffic conditions is essential, even usually more important that the sleep period itself. The application of the source coding in ODYSSE improves reliability at the expense of a low energy cost. We argue that, to maximize the gain from source coding (through higher code rate), an optimized implementation of these codes is mandatory.

## Figures and Tables

**Table 1:** Experiment parameters.

ACTIVE_PERIOD	0.2 s	WAIT_DATA_RPERIOD	3 s
MIN_SLEEP_PERIOD	0.05 s	RSSI_THRESHOLD	−83 dBm
LEVEL_PERIOD	8 s	BEACON_PERIOD	3 s
MAX_NB_REPLY	1	SHORT_SLEEP_COUNT	3
WAIT_REPLY_PERIOD	0.2 s		

## Appendix A. Flowchart of ODYSSE

Figure A1 illustrates the operations of ODYSSE.
